# The association between anterior cruciate ligament degeneration and incident knee osteoarthritis: Data from the osteoarthritis initiative

**DOI:** 10.1016/j.jot.2023.09.005

**Published:** 2023-12-14

**Authors:** Ping Luo, Qianyi Wang, Peihua Cao, Tianyu Chen, Shengfa Li, Xiaoshuai Wang, Yamin Li, Ze Gong, Yan Zhang, Guangfeng Ruan, Zuoqing Zhou, Yuanyuan Wang, Weiyu Han, Zhaohua Zhu, David J. Hunter, Jia Li, Changhai Ding

**Affiliations:** a Clinical Research Centre, Zhujiang Hospital, Southern Medical University, Guangzhou, Guangdong, China; b Division of Orthopaedic Surgery, Department of Orthopedics, Nanfang Hospital, Southern Medical University, Guangzhou, Guangdong, China; c Department of Spinal Surgery, The Fourth Hospital of Changsha, Changsha Hospital of Hunan Normal University, Changsha, Hunan, China; d Department of Orthopedics, The Third Affiliated Hospital of Southern Medical University, Guangzhou, China; e Department of Nephrology, Guangzhou First People's Hospital, School of Medicine, South China University of Technology, Guangzhou, China; f Department of Rehabilitation Medicine, Zhujiang Hospital, Southern Medical University, Guangzhou, China; g Clinical Research Centre, Guangzhou First People's Hospital, School of Medicine, South China University of Technology, Guangzhou, China; h Department of Orthopedics, The First Affiliated Hospital, Shaoyang University, Shaoyang, Hunan, China; i Department of Health Management, Nanfang Hospital, Southern Medical University, Guangzhou, Guangdong, China; j Department of Rheumatology, Royal North Shore Hospital and Sydney Musculoskeletal Health, Kolling Institute, University of Sydney, Australia; k Menzies Institute for Medical Research, University of Tasmania, Hobart, Tasmania, Australia

**Keywords:** Anterior cruciate ligament, Degeneration, Knee osteoarthritis, MRI

## Abstract

**Background:**

Though anterior cruciate ligament (ACL) tear has been widely accepted as an important accelerator for knee osteoarthritis (KOA), the role of intrinsic ACL degeneration in developing KOA has not been fully investigated.

**Purpose:**

To determine whether ACL degeneration, in the absence of ACL tear, is associated with incident KOA over 4 years.

**Study design:**

Cohort study; Level of evidence, 2.

**Methods:**

Participants’ knees in this nested case–control study were selected from the Osteoarthritis Initiative (OAI) study, with Kellgren–Lawrence grading (Kellgren–Lawrence grading) of 0 or 1 ​at baseline (BL). Case knees which had incident KOA (KLG ≥2) over 4 years, were matched 1:1 with control knees by gender, age and radiographic status. ACL signal intensity alteration (0–3 scale) and volume were assessed as compositional feature and morphology of ACL degeneration, using knee MRI at P0 (time of onset of incident KOA), P-1 (1 year prior to P0) and baseline. Conditional logistic regression was applied to analyze the association between measures of ACL degeneration and incident KOA.

**Results:**

337 case knees with incident KOA were matched to 337 control knees. Participants were mostly female (68.5%), with an average age of 59.9 years old. ACL signal intensity alterations at BL, P-1 and P0 were significantly associated with an increased odds of incident KOA respectively (all *P* for trend ≤0.001). In contrast, ACL volumes were not significantly associated with incident KOA at any time points.

**Conclusions:**

ACL signal intensity alteration is associated with increased incident KOA over 4 years, whereas ACL volume is not.

**The translational potential of this article**: This paper focused on ACL signal intensity alteration which could better reflect ACL degeneration rather than ACL tear during the progression of KOA and explored this topic in a nested case–control study. Utilizing MR images from KOA participants, we extracted the imaging features of ACL. In addition, we established a semi-quantitative score for ACL signal intensity alteration and found a significant correlation between it and KOA incidence. Our findings confirmed that the more severe the ACL signal intensity alteration, the stronger relationship with the occurrence of KOA. This suggests that more emphasis should be placed on ACL degeneration rather than ACL integrity in the future.

## Graphical abstract

**Figure:**
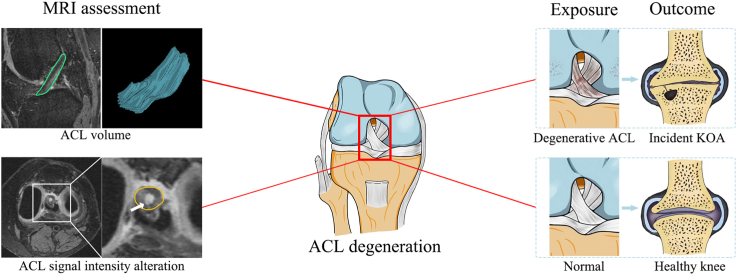

## 1 Introduction

Knee osteoarthritis (KOA) is one of the most important causes of disability, which greatly increases societal burden in the elderly [1]. As a whole joint disease, structural changes, including cartilage degradation, subchondral bone marrow lesion (BML), synovitis and ligament degeneration, are thought to occur in the context of KOA development [2]. The anterior cruciate ligament (ACL) serves as the main static and dynamic stabilizer to maintain knee stability, but the potential role of ACL degeneration has been poorly understood in KOA.

ACL tear has been widely accepted as an important accelerator for knee structural damage [[3], [4], [5], [6]]. Most ACL tears occur in athletes, with an overall incidence of only 1 in 3500 people in the general population [7]. For most individuals without traumatic factors; however, the potential role of intrinsic ACL degeneration has not been well recognized. Various compositional features of ACL degeneration have been reported in histologic study, including collagen fiber disorganization, mucoid degeneration, cyst formation and chondroid metaplasia [8]. Meanwhile, ACL morphology (i.e., volume and maximum area), which may alter during the process of KOA, has been reported to be associated with age and gender [[9], [10], [11]].

Nevertheless, as a vital structure within the capsule, the general role of intrinsic ACL degeneration in the development of KOA remains elusive. Only a few studies investigated the compositional feature of ACL degeneration in the development of KOA. Kwee et al. reported that ACL mucoid degeneration was associated with both cartilage damage [5] and joint space loss (JSL) [4] in medial tibiofemoral compartment. Gersing et al. also reported that ACL mucoid degeneration was associated with higher progression rates of cartilage degeneration compared with normal ACL [3]. Roemer et al. used a semi-quantitative scoring system of the Anterior Cruciate Ligament OsteoArthritis Score (ACLOAS) to evaluate ACL signal alteration on MRI, but focused on acute ACL injury rather than chronic ACL degeneration [12,13]. Kiapour et al. attempted to assess both signal intensity and cross-sectional area of healing ACL or grafts after surgery, providing a direct clue of tissue quality and morphology [14]. Up to date, the general quality and morphology of ACL degeneration have not been well studied in the development of KOA and thus the findings are inconclusive.

The purpose of this nested case–control study was to explore the association between ACL degeneration and incident KOA in the Osteoarthritis Initiative (OAI) study. We hypothesize that both compositional feature and morphology of ACL degeneration lead to an increased risk of KOA incidence.

## 2 Methods

### 2.1 Study design and participants

All data in the study were obtained from the OAI, a multicenter, prospective cohort study on KOA. 4796 participants were initially recruited from February 2004 to May 2006 ​at four clinical sites in the United States. The Institutional Review Board (IRB) at each of the sites approved the study, with written informed consent obtained from all participants. All datasets analyzed during the study are available at https://nda.nih.gov/oai/.

**Figure 1:**
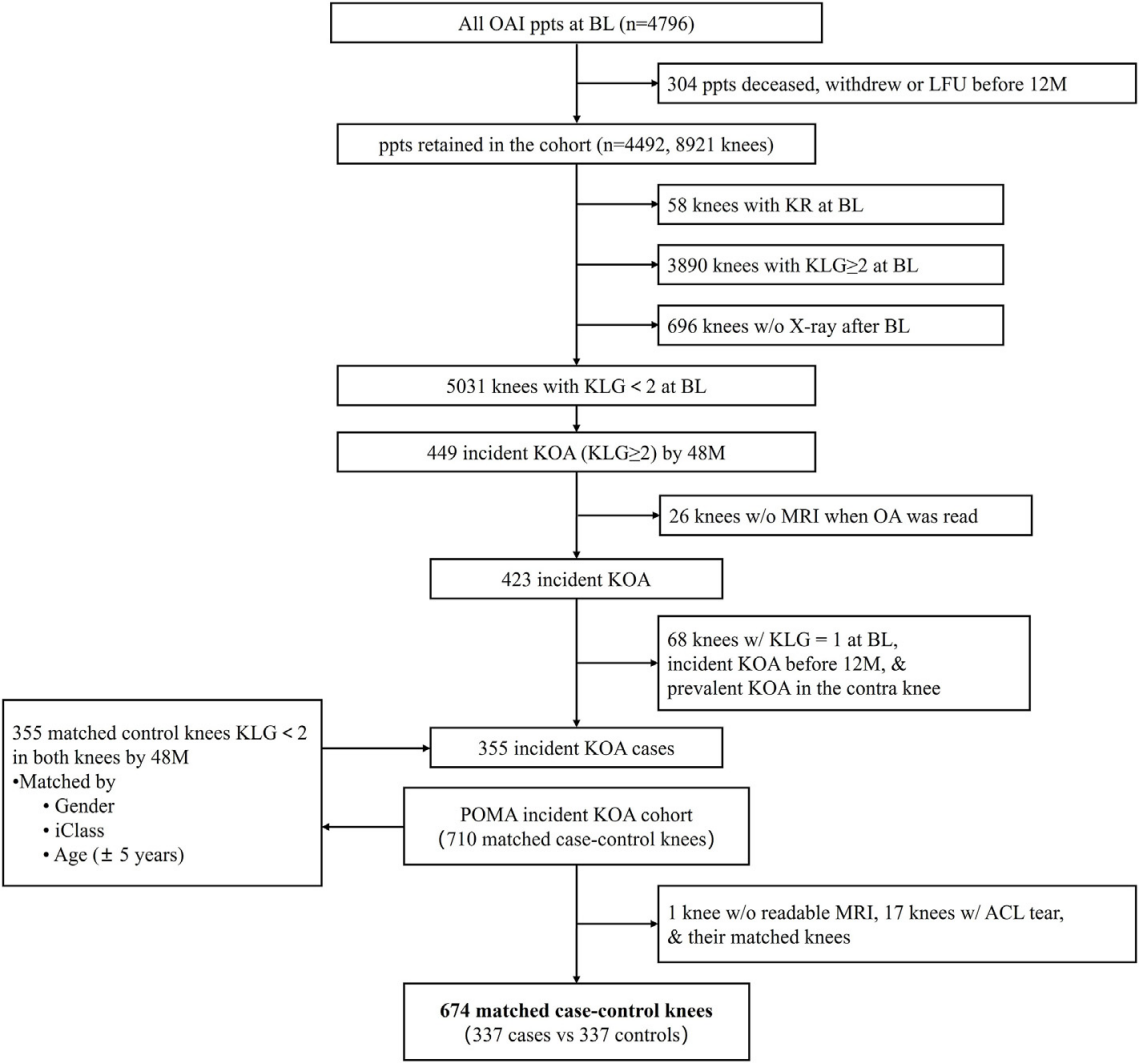
Flowchart showing inclusion of study knees. iClass refers to contralateral radiographic status (i.e. KLG of 0, 1 or 2+ in the contralateral knee). OAI = Osteoarthritis Initiative; ppt ​= ​participant; BL ​= ​baseline; LFU ​= ​loss of follow-up; M ​= ​month; KR ​= ​knee replacement; KLG = Kellgren Lawrence grading; KOA ​= ​knee osteoarthritis; MRI ​= ​magnetic resonance imaging; w/o ​= ​without; w/= with.

We used the incident KOA cohort from the Pivotal OAI MR Imaging Analysis (POMA) study, which consisted of 710 knees selected for a nested, 1-to-1 matched case–control analysis [15]. 17 knees with both partial and complete ACL tears and 1 knee without readable MRI sequence were excluded from the sample of POMA, along with their matching knees; therefore, a total of 674 knees were included in the final analysis (Fig. 1). All participants were 45–79 years old without KOA, but at increased risk of developing KOA with the presence of more than 2 of the following risk factors: overweight, knee injury or surgery history, family history of knee replacement, or Heberden's nodes. Cases were defined as knees that progressed to Kellgren–Lawrence grading (KLG) ≥ 2 by 48 months, while controls were defined as knees that did not have or progress to KLG ≥ 2 by that time [16].

### 2.2 Radiography

Fixed-flexion radiography was performed in both case and control knees at baseline and all annual follow-up visits. Bilateral, standing posteroanterior (PA) knee films were obtained, with knees flexed 20–30° and feet internally rotated 10°. Knee radiographs were read by central readers to assess for the KLG, with KOA defined as KLG ≥ 2.

### 2.3 Cases and controls

The case knee had KLG ≤1 ​at baseline, and developed KOA at any annual visit during the follow-up of 48 months. The control knee also had KLG ≤1 ​at baseline, but did not develop KOA within 48 months. Each case knee was matched 1:1 with a control knee by gender, age (±5 years), and contralateral radiographic status (i.e. KLG of 0, 1 or 2+ in the contralateral knee). P0 was defined as the first incident time point of KOA, with P-1 being defined as the time point 1 year before incident KOA was detected.

There was a total of 710 knees in the original POMA incident KOA cohort. 674 knees were finally included in this study after excluding knees with ACL tears, with annual fixed-flexion radiography and MRI from baseline to 48 months.

Baseline knee injury and knee surgery histories were recorded during the enrollment visit by asking the participants if they had ever experienced a knee injury that resulted in difficulty walking for at least a week, or if they had ever undergone any type of knee surgery, including ligament repair, arthroscopy, or meniscectomy. Isometric knee extensor and flexor strength (in newtons) were measured by OAI personnel, using a Good Strength Chair (Metitur Oy) [17]. Additionally, during the same visit, participants’ physical examinations of body mass index (BMI) and knee alignment (normal, varus and valgus) were taken.

### 2.4 MRI assessment

MRIs of target knees were performed using identical 3.0 ​T instruments (Trio, Siemens Healthcare, Erlangen, Germany) at four OAI clinical centers, with a dedicated quadrature transmit/receive knee coil. Non-contrast enhanced sequences were obtained with previously reported protocols [16]. Among them, sagittal intermediate-weighted (IW) fat saturated (FS) turbo spin-echo sequence, coronal IW turbo spin-echo sequence, sagittal T2-weighted 3D dual-echo in steady-state (DESS) sequence and its axial and coronal multiplanar reformats (MPRs) were applied in this study. Both compositional feature and morphology of ACL degeneration were performed at BL (baseline), P-1 and P0 using the software program Osirix (University of Geneva). All MRIs were read in a consecutive manner by an experienced orthopedist (PL), unblinded to the time point but blinded to the status of case/control.

The presence of an ACL tear at baseline was detected by sagittal and coronal views of IW turbo spin-echo sequences and scored on a 0–2 scale (0 ​= ​intact, 1 ​= ​partial tear and 2 ​= ​complete tear). ACL complete tear was defined as complete disruption of ACL fibres with ligament discontinuity, while ACL partial tear was defined as residual straight and tight ligament fibre in at least one-pulse sequence [18]. As mentioned above, knees with both partial and complete ACL tears were all excluded in this study, avoiding the interference of traumatic factors. All MRIs were read by experienced orthopedists (PL and YL) and reached an agreement before the exclusion.

**Figure 2:**
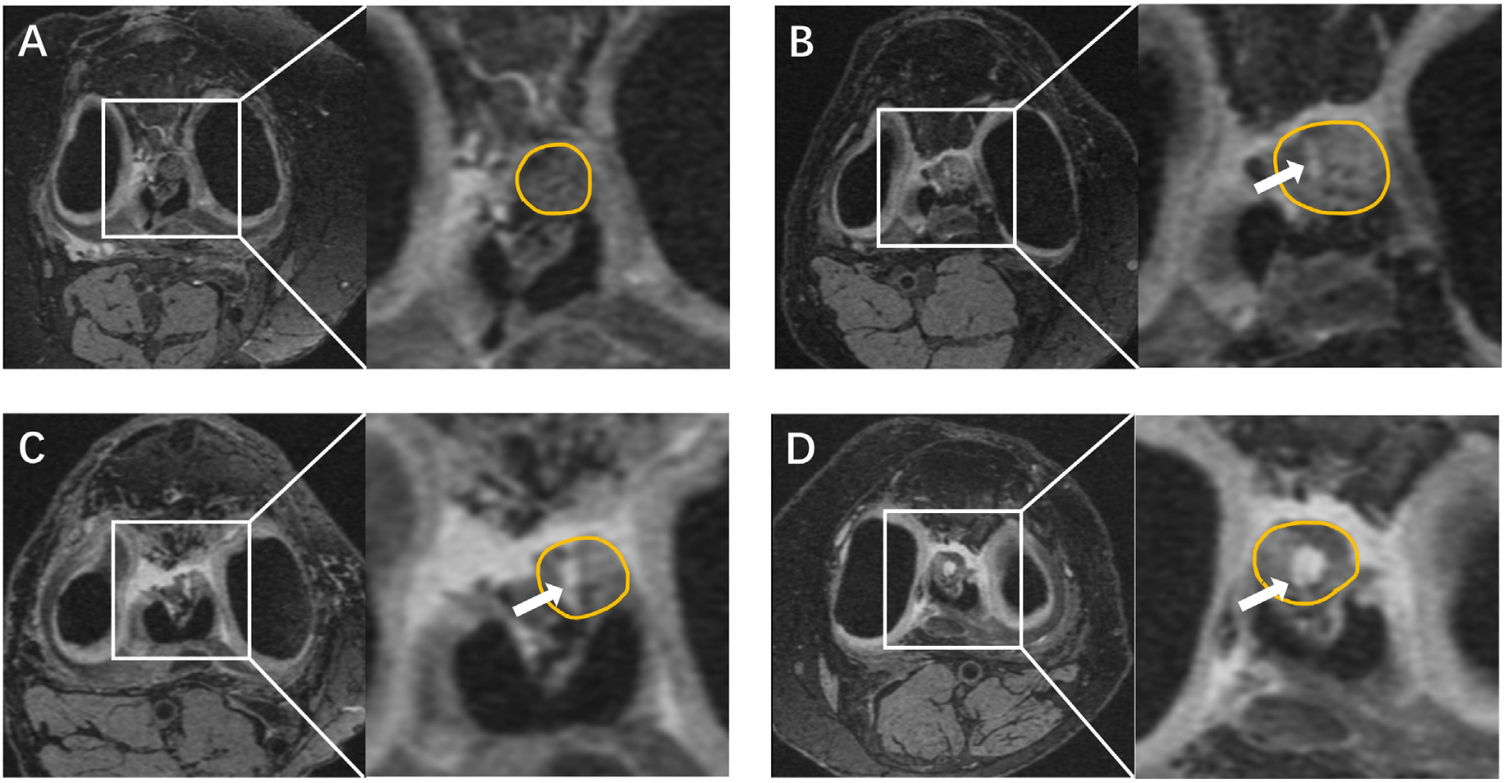
ACL signal intensity alteration: A. normal ACL. B. arrow indicates signal intensity alteration of ACL (grade 1 ​≤ ​10% of the region). C. arrow points to signal intensity alteration of ACL (grade 2 ​= ​10%–20% of region). D. arrow indicates signal intensity alteration of ACL (grade 3 ​> ​20% of the region).

ACL signal intensity alteration, defined as discrete signal intensity alteration within the ligament on axial T2-weighted 3D-DESS reformat. The slice with maximum high signal intensity area was selected to represent ACL signal intensity alteration. The percentage affected with the largest area of high signal intensity in the total ACL area was used to assess the level of ACL signal intensity alteration. Inspired by similar studies on other tissue [19,20], it was finally classified into four grades: grade 0 ​= ​none; grade 1 ​≤ ​10% of ACL region; grade 2 ​= ​10%–20% of ACL region; grade 3 ​≥ ​20% of ACL region (Fig. 2), after reviewing all MRIs in this study. The intra-observer and inter-observer reliabilities (performed by PL and YL) were assessed in 30 randomly selected knees from the whole sample, with a weighted κ of 0.97 (95%CI 0.92, 1.00) and 0.90 (95%CI 0.77, 1.00), respectively.

**Figure 3:**
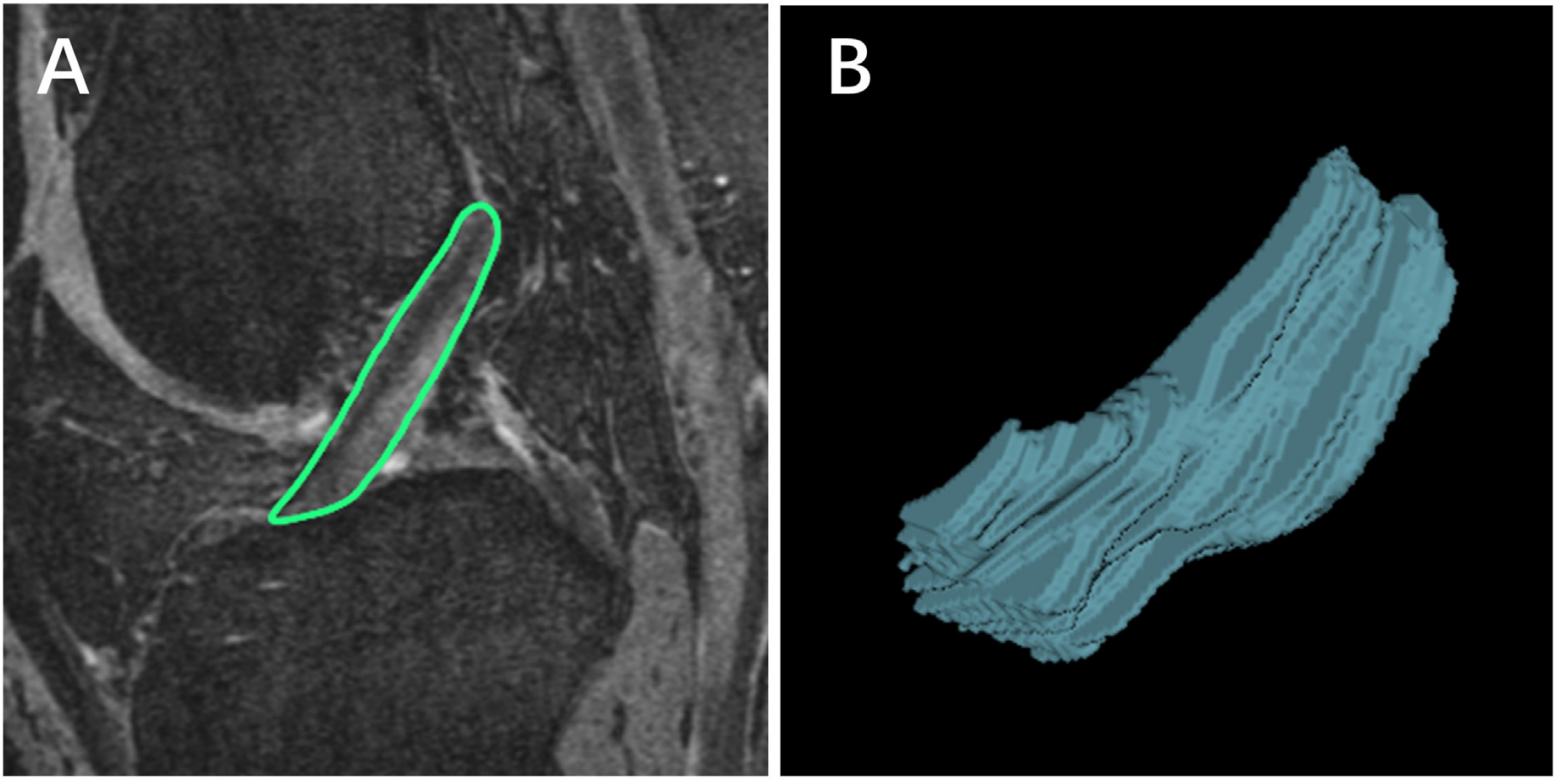
ACL volume: A. Manual delineation of ACL contour on each image. B. ACL volume was calculated by summiting up the area of each slice multiplied by the slice thickness, using Osirix.

ACL volume was measured on sagittal T2 weighted 3D-DESS images. Approximately 15–20 images with ACL boundary were selected from a total of 160 ​MR images. ACL volume was measured by manually drawing disarticulation contours around the ACL boundaries on section-by-section sagittal T2 weighted 3D-DESS images, using the software program Osirix. ACL volume was then computed by the software program to represent ACL size (Fig. 3). The intra-observer and inter-observer reliabilities (performed by PL and YL) were assessed in 30 randomly selected knees, with an intra-class correlation coefficient (ICC) of 0.95 (95% CI 0.90, 0.98) and 0.94 (95%CI 0.89, 0.97), respectively.

Considering the potential effect of anatomic variations in the physical size differences of participants’ knees, the notch width index (NWI) was applied to accurately reflect the width and stenosis of the intercondylar notch [21]. NWI was measured on coronal MPRs, and determined as the ratio of the femoral intercondylar width to that of the internal and external condyles at the level of the popliteal tendon.

### 2.5 Statistical analysis

The *chi*-square and pair-*t* tests were used to compare the proportions and means between case and control groups. Conditional logistic regression models were used to analyze the associations between compositional feature and morphology of ACL degeneration and incident KOA. General estimated equations (GEEs) were applied to account for the correlation between two knees within the same individual. Models were firstly run without any adjustment, and other two models were run with adjustments for baseline covariates, i.e. one included history of knee injury, knee surgery, BMI, gender and ethnicity, and the other included the covariates mentioned above and thigh muscle strength, knee alignment and NWI. Models were run for ACL signal intensity alteration and ACL volume, respectively, at three time-points: P0 (concurrent with incident radiographic KOA), P-1 (1 year prior to incident KOA) and baseline. All analyses were performed in Stata/SE 15.1 (StataCorp). A *P* value less than 0.05 (2-tailed) was considered statistically significant.

## 3 Results

The demographics and baseline characteristics of case and control knees are summarized in Table 1. Participants were predominantly female (68.5%), with an average age of 59.8 ​± ​8.4 (mean ​± ​SD) years old. There were 110 case knees (32.6%) at 12 months, 80 (23.7%) at 24 months, 98 (29.1%) at 36 months, and 49 (14.6%) at 48 months, as well as control knees. No significant differences were found in terms of age, gender, ethnicity and previous knee surgery, but case knees had higher mean BMI and a higher percentage of previous knee injury.

**Table 1:** Baseline characteristics of the case and control knees in the study population.

Characteristics	Total(n ​= ​674)	Control knees (n ​= ​337)	Case knees (n ​= ​337)	*P* value
Female, N (%)	462 (68.5)	231 (68.5)	231 (68.5)	>0.99
Age, mean ​± ​SD	59.9 ​± ​8.4	59.9 ​± ​8.4	59.9 ​± ​8.6	0.864
Ethnicity, N (%)				0.306
White or Caucasian	552 (81.9)	283 (84.0)	269 (79.8)	
Other	122 (18.1)	54 (16.0)	68 (20.2)	
Knee injury, N (%)	**141 (20.9)**	**58 (17.2)**	**83 (24.6)**	**0.018**
Knee surgery, N (%)	35 (5.2)	20 (5.9)	15 (4.5)	0.385
BMI, mean ​± ​SD	**28.4** ​± ​**4.5**	**27.7** ​± ​**4.4**	**29.0**± ​**4.6**	**< 0.001**
Incidence timepoint, N (%)				>0.99
12th month	220 (32.6)	110 (32.6)	110 (32.6)	
24th month	160 (23.7)	80 (23.7)	80 (23.7)	
36th month	196 (29.1)	98 (29.1)	98 (29.1)	
48th month	98 (14.5)	49 (14.5)	49 (14.5)	
Contralateral radiographic status (KLG in index knee/KLG in contralateral knee), N (%)				>0.99
1 (0/0)	116 (17.2)	58 (17.2)	58 (17.2)	
2 (0/1)	148 (22.0)	74 (22.0)	74 (22.0)	
3 (1/1)	156 (23.1)	78 (23.1)	78 (23.1)	
4 (0/2+)	114 (16.9)	57 (16.9)	57 (16.9)	
5 (1/2+)	140 (20.8)	70 (20.8)	70 (20.8)	
Knee alignment, N (%)				0.389
Normal	200 (29.7)	109 (32.3)	91 (27.0)	
Varus	171 (25.3)	78 (23.1)	93 (27.6)	
Valgus	303 (45.0)	150 (44.5)	153 (45.4)	
Muscle strength, mean ​± ​SD				
Extensor strength	344.2 ​± ​125.0	339.2 ​± ​122.3	348.9 ​± ​127.5	0.408
Flexor strength	140.1 ​± ​62.1	142.1 ​± ​61.2	138.2 ​± ​63.0	0.432
NWI (%), mean ​± ​SD	26.3 ​± ​2.6	26.2 ​± ​2.6	26.4 ​± ​2.7	0.470

KLG: Kellgren Lawrence grading; BMI: body mass index; NWI: notch width index.

Bold denoted statistical significance.

**Figure 4:**
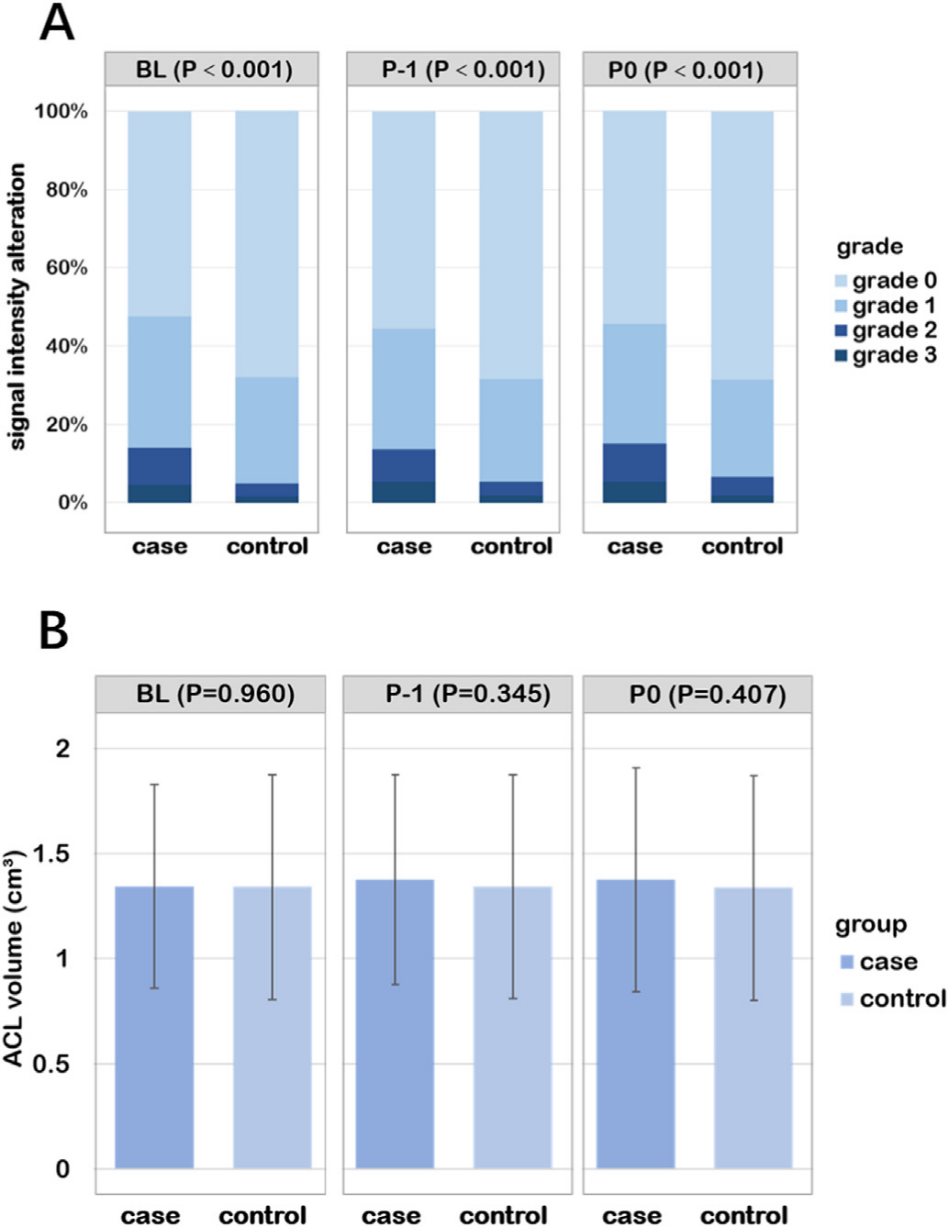
A. Comparison of ACL signal intensity alteration between case and control groups using accumulative bar charts; Wilcoxon rank-sum tests were used for calculating *P* value; accumulative bars show percentages for ACL signal intensity alteration. B. Comparison of ACL volume between case and control groups using bar charts; Paired-Samples t tests were used for calculating *P* value; bars show mean ​± ​SD. P0: the visit when incident KOA was observed on radiograph; P-1: 1 year prior to P0; BL: baseline.

Comparison of all measures of ACL between cases and controls are presented in Fig. 4. The case group had higher percentages of ACL signal intensity alteration (including grade 1, grade 2 and grade 3) than control group at baseline, P-1 and P0 (Fig. 4-A), but there were no significant differences of ACL volume between case and control groups at three time points (Fig. 4-B).

Associations between ACL signal intensity and subsequent incident KOA as shown in Table 2. Both in unadjusted and adjusted analyses, ACL signal intensity alterations were significantly associated with increased odds of incident KOA at three time points (all *P* for trend ≤0.001). In the multivariable model, grade 1, 2 and 3 of ACL signal intensity alteration had 1.69-fold, 3.72-fold and 4.66-fold increased odds of incident KOA at BL, respectively, compared to grade 0. Similarly, grade 1, 2 and 3 of ACL signal intensity alteration had 1.65-fold, 2.59-fold and 4.12-fold increased odds of incident KOA at P-1, and had 1.54-fold, 2.72-fold and 3.90-fold increased odds at P0, respectively, compared to grade 0. The results remained largely unchanged after further adding thigh muscle strength, knee alignment and NWI as potential confounders.

**Table 2:** Association between ACL signal intensity alteration and incident KOA at BL, P-1, and P0.

ACL signal intensity alteration	ORs^a^ (95% CI)	*P* value^a^	Adjusted ORs^b^ (95% CI)	*P* value^b^	Adjusted ORs^c^ (95% CI)	*P* value^c^
**BL (n** ​= ​**674)**						
**0**	Reference		Reference		Reference	
**1**	**1.59(1.12,2.25)**	**0.009**	**1.69(1.19,2.40)**	**0.004**	**1.92(1.31,2.80)**	**0.001**
**2**	**3.76(1.83,7.72)**	**<0.001**	**3.72(1.81,7.64)**	**<0.001**	**3.25(1.56,6.78)**	**0.002**
**3**	**3.88(1.38,10.91)**	**0.010**	**4.66(1.69,12.89)**	**0.003**	**4.17(1.44,12.07)**	**0.008**
*P* for trend		**<0.001**		**<0.001**		**<0.001**
**P-1 (n** ​= ​**651)**						
**0**	Reference		Reference		Reference	
**1**	**1.54(1.08,2.21)**	**0.018**	**1.65(1.15,2.38)**	**0.007**	**1.82(1.24,2.67)**	**0.002**
**2**	**2.53(1.33,4.82)**	**0.005**	**2.59(1.39,4.83)**	**0.003**	**2.00(1.03,3.86)**	**0.039**
**3**	**3.83(1.48,9.91)**	**0.006**	**4.12(1.52,11.17)**	**0.005**	**3.30(1.13,9.64)**	**0.029**
*P* for trend		**<0.001**		**<0.001**		**<0.001**
**P0 (n** ​= ​**654)**						
**0**	Reference		Reference		Reference	
**1**	**1.46(1.02,2.09)**	**0.037**	**1.54(1.07,2.20)**	**0.019**	**1.63(1.11,2.38)**	**0.012**
**2**	**2.84(1.38,5.84)**	**0.004**	**2.72(1.33,5.57)**	**0.006**	2.14 (1.00,4.57)	0.050
**3**	**3.72(1.43,9.64)**	**0.007**	**3.90(1.49,10.23)**	**0.006**	**3.29(1.14,9.48)**	**0.027**
*P* for trend		**<0.001**		**<0.001**		**0.001**

ACL: anterior ligament cruciate; KOA: knee osteoarthritis; BMI: body mass index; NWI: notch width index; P0: the visit when incident KOA was observed on radiograph; P-1: 1 year prior to P0; BL: baseline.

Bold denoted statistical significance.

a Univariable conditional logistic regression without any adjustment.

b Adjustment for self-reported knee injury, self-reported knee surgery, BMI, gender and ethnicity.

c Adjustment for self-reported knee injury, self-reported knee surgery, BMI, gender, ethnicity, NWI, knee alignment, extensor strength and flexor strength.

Associations between ACL volume and incident KOA at any of the three time points are shown in Table 3. ACL volumes were not significantly associated with incident KOA at all three time points, both in unadjusted and adjusted analyses. The ACL maximum area was also analyzed and obtained similar results (Supplementary Table 1).

**Table 3:** Association between ACL volume and incident KOA at BL, P-1, and P0.

ACL volume	ORs^a^ (95% CI)	*P* value^a^	Adjusted ORs^b^ (95% CI)	*P* value^b^	Adjusted ORs^c^ (95% CI)	*P* value^c^
**BL (n** ​= ​**674)**						
	1.01 (0.74,1.38)	0.962	0.98 (0.65,1.46)	0.912	1.00 (0.64,1.56)	>0.99
**P-1 (n** ​= ​**651)**						
	1.13 (0.83,1.55)	0.429	1.14 (0.75,1.73)	0.552	1.15 (0.75,1.75)	0.529
**P0 (n** ​= ​**654)**						
	1.15 (0.84,1.56)	0.374	1.16 (0.77,1.74)	0.473	1.23 (0.82,1.85)	0.323

ACL: anterior ligament cruciate; KOA: knee osteoarthritis; BMI: body mass index; NWI: notch width index; P0: the visit when incident KOA was observed on radiograph; P-1: 1 year prior to P0; BL: baseline.

Bold denoted statistical significance.

a Univariable conditional logistic regression without any adjustment.

b Adjustment for self-reported knee injury, self-reported knee surgery, BMI, gender and ethnicity.

c Adjustment for self-reported knee injury, self-reported knee surgery, BMI, gender, ethnicity, NWI, knee alignment, extensor strength and flexor strength.

## 4 Discussion

To the best of our knowledge, it is the first study to focus on the associations between compositional feature and morphology of ACL degeneration and incident KOA in a specific population without ACL tears. We found that ACL signal intensity alteration was significantly associated with increased incident KOA during 4 years, while ACL volume had non-significant associations. These results suggest that compositional feature of ACL degeneration, rather than morphology, plays a role in the development of KOA.

ACL, which connects the inner surface of lateral femoral condyle with the anterior intercondylar area of tibia [22], is localized intra-articularly and exerts a stabilizing function during the knee movement [23]. It has been well acknowledged that people sustaining an ACL tear have an increased risk of developing KOA [24]. However, the association of ACL degeneration with incident KOA remains poorly investigated. As we know, an ACL tear is a relatively infrequent event in the general population. Some individuals without prior history of ligament injury may suffer intrinsic ACL degeneration as well. It has been reported that the histologic score of ACL degeneration was correlated with KOA in a cross-sectional study of end-staged KOA with a small sample size [25]; however, it is unknown whether ACL degeneration observed in end-stage KOA is a cause or consequence of KOA. The temporal relationship between ACL degeneration and incident KOA is still largely unknown.

MRI is an important tool in assessing intra-articular soft tissues, but its application is mainly limited to detecting partial or complete ACL tears when diagnosing KOA [3,26]. ACL degeneration has been rarely analyzed through MRI. By using MRI, it has been previously reported that ACL mucoid degeneration was associated with both cartilage damage [5] and joint space loss (JSL) [4] in medial tibiofemoral compartment. Gersing et al. also reported that ACL abnormalities, including mucoid degenerations and tears, were associated with higher progression rates of cartilage degeneration compared with normal ACL [3]. Nevertheless, the other compositional features of ACL degeneration, which had been reported in previous histologic studies (i.e. collagen fiber disorganization, inflammation, cystic changes and chondroid metaplasia) [8,27,28], were not taken into account and thus led to incomplete conclusions.

Our study measured all ACL compositional features of ACL degeneration as a whole and used semi-quantitative assessment regarding its affected areas in axial reformatting sequence for the first time. Previous studies have proposed that sagittal imaging in combination with axial one could better detect ACL degeneration, suggesting the value of axial imaging in ACL diagnosis. Because the ACL is composed of multiple fiber bundles constituted by collagens [2,29], which differs from other ligaments by less compact collagen architecture, higher glycosaminoglycan and elastin contents, and makes intrinsic abnormalities spread along the interfasciculum [30]. So we used axial imaging to better exhibit the severity of compositional change of ACL degeneration in this study, compared with sagittal one. Given the potential influence of ACL injury, we excluded individuals with partial or complete ACL tears. As for the measurement, we failed to quantitatively measure the area affected by signal intensity alteration in ACL, regarding the small and discrete affected area with unclear contours. Besides, there were also individual differences concerning the total size of ACL, which seemingly made semi-quantitative assessment of affected percentage more reasonable. We found that ACL signal intensity alteration was associated with incident KOA in 4 years, using a nested case–control design. To our knowledge, no studies have focused on the compositional feature of ACL degeneration in such a specific population without ACL tears. Previously, ACL tears would interfere with the results when looking at the impact of feature abnormalities of ACL. Our study provided further evidence emphasizing the compositional feature of ACL degeneration in the general population.

On the other hand, the compositional feature of ACL degeneration may accompany with the morphology. Previous studies have explored ACL morphology in the young population. For example, Wang et al. reported that men had greater ACL volume than women, which can explain why ACL injuries were more prone to occur in women than in men [31]. Iriuchishima et al. found the sagittal ACL area seemed to be larger in youngsters compared with the elderly [10], indicating that ACL size may alter during the aging process. But there have been no studies investigating the association between ACL morphology and incident KOA. It was the first exploration to determine the role of ACL morphology in KOA development, though we did not find any significant associations of ACL volume with incident KOA. The potential explanation could be that it is ACL compositional feature rather than morphology leading to incident KOA.

The exact pathogenesis of ACL degeneration remains poorly understood, which may be triggered by long-term chronic wear. Histologically, ACL degeneration was characterized by recruitment or proliferation of cells, including myofibroblasts and progenitor cells [32]. Compared with other ligaments, ACL is a double-bundle structure, with less compact collagen architecture and higher glycosaminoglycan and elastin. Collagen fiber disorganization and mucoid degeneration, characterized by hypertrophy and deposition of glycosaminoglycan amidst the collagen bundles [33], are thought to be the earliest detectable abnormalities during ACL degradation [8]. Different compositional features of ACL degeneration always coexist in KOA, so it could be a better way to evaluate the general status of affected areas within ACL, instead of defining each compositional feature separately.

The potential mechanism for the impact of ACL degeneration on KOA remains to be elucidated. ACL has an inferior healing capability as a result of poor vascularization and microstructural irregularities, leading to impairing stabilizing function in a mechanical way during the knee movement [32,34]. Moreover, ACL inflammation manifests as neovascularization and leukocyte infiltration in the ligament sheath and within the ACL substance, which may induce adjacent cartilage degeneration [8]. As a whole, we deduce that both mechanical and chemical processes mentioned above may be involved in the development of KOA.

The main strength of the study is that both compositional feature and morphology of ACL degeneration were assessed prior to the incidence of KOA in a well-designed nested case–control study. Nevertheless, there were also several potential limitations. First, the MR images were not acquired from an oblique sagittal-plane, which can display a clearer profile and texture of ACL. Therefore, we applied axial MPRs to assess ACL signal intensity alteration, and delineated the ACL contour at multiple slices to calculate the volume. Second, pathological examinations were unable to be implemented in our study so the pathological changes associated with ACL degeneration were unknown. Third, there may exist confounding effects in the light of knee injury, self-report surgery, BMI, gender, ethnicity, thigh muscle strength, knee alignment and NWI at baseline, which may influence the results; however, the results remained statistically significant after adding these factors as potential confounders into multivariable models. Fourth, this study was a nested case–control design with only one outcome, so we failed to explore the association between the severity of ACL degeneration and the severity of KOA. The rapid incidence of KOA over 4 years may also have a potential impact on the results. Therefore, longer follow-up time points should be launched in a more specific longitudinal design. Fifth, this study used only X-rays to diagnose KOA, which is not as effective as MRI for detection of early-stage KOA. The current gold standard for KOA diagnosis is still X-rays. Further study including MRI data should be developed in the future.

## 5 Conclusions

The compositional feature of ACL degeneration, rather than morphology, is associated with increased incident KOA over 4 years. Further clinical and pathological studies are required to verify the causal relationships.

## Funding

This work was supported by the 10.13039/501100001809National Natural Science Foundation of China (82103903, 81974342, 82102622), Guangdong Basic and Applied Basic Research Foundation (2021A1515010005), President Foundation of 10.13039/501100010112Nanfang Hospital, 10.13039/501100010096Southern Medical University (2021C006) and the Natural Science Foundation of Hunan Province of China (2021JJ40518). The study and image acquisition were funded by the OAI, a public-private partnership comprised of five contracts (N01-AR-2- 2258; N01-AR-2-2259; N01-AR-2-2260; N01-AR-2-2261; N01-AR- 2–2262) funded by the 10.13039/100000002National Institutes of Health, a branch of the 10.13039/100000002Department of Health and Human Services, and conducted by the OAI Study Investigators. Private funding partners of the OAI include 10.13039/100004334Merck Research Laboratories; 10.13039/100008272Novartis Pharmaceuticals Corporation, GlaxoSmithKline; and 10.13039/100004319Pfizer, Inc. Private sector funding for the OAI is managed by the Foundation for the 10.13039/100000002National Institutes of Health. The image analysis of this study was funded by a contract with the 10.13039/100007921University of Pittsburgh (Pivotal OAI 10.13039/100011612MRI Analyses POMA: NIH/10.13039/100000050NHLBI Contract No. HHSN2682010000 21C), and in part by a vendor contract from the OAI coordinating center at University of California, San Francisco (N01-AR-2-2258). None of the study sponsors had any role in data collection, storage or analysis, in manuscript writing, or the decision to publish this manuscript.

## Declaration of competing interest

The authors have no professional or financial affiliations that may be perceived to have biased the presentation.
