# Supplementary material for: The association between anterior cruciate ligament degeneration and incident knee osteoarthritis: Data from the osteoarthritis initiative
Source: J Orthop Translat. 2023 Dec 14;44:1–8. doi: 10.1016/j.jot.2023.09.005 (PMC10762318; doi:10.1016/j.jot.2023.09.005)
Supplement: Multimedia component 1 [file mmc1.docx]

**Supplementary Table 1.** Association between ACL maximum area and incident KOA at BL, P-1, and P0.

| ACL maximum area | ORs^*^  (95% CI) | *P* value^*^ |  | Adjusted ORs**^ƚ^**  (95% CI) | *P* value**^ƚ^** |  | Adjusted ORs^ʄ^  (95% CI) | *P* value^ʄ^ |
| --- | --- | --- | --- | --- | --- | --- | --- | --- |
| **BL (n=674)** |  |  |  |  |  |  |  |  |
|  | 1.05(0.79,1.40) | 0.727 |  | 1.08(0.78,1.50) | 0.642 |  | 1.05(0.74,1.49) | 0.793 |
| **P-1 (n=651)** |  |  |  |  |  |  |  |  |
|  | 1.25(0.94,1.66) | 0.123 |  | 1.31(0.94,1.82) | 0.109 |  | 1.26(0.89,1.77) | 0.187 |
| **P0 (n=654)** |  |  |  |  |  |  |  |  |
|  | 1.26(0.96,1.67) | 0.100 |  | 1.31(0.95,1.81) | 0.098 |  | 1.35(0.97,1.90) | 0.078 |

ACL: anterior ligament cruciate; KOA: knee osteoarthritis; BMI: body mass index; NWI: notch width index; P0: the visit when incident KOA was observed on radiograph; P-1: 1 year prior to P0; BL: baseline.

Bold denoted statistical significance.

^*^Univariable conditional logistic regression without any adjustment.

^ƚ^Adjustment for self-reported knee injury, self-reported knee surgery, BMI, gender and ethnicity.

^ʄ^Adjustment for self-reported knee injury, self-reported knee surgery, BMI, gender, ethnicity, NWI, knee alignment, extensor strength and flexor strength.

Derived from ACL volume, ACL maximum area was selected from 15-20 MR images with ACL boundary.
